# Supplementary material for: CRISPRi-Guided Metabolic Flux Engineering for Enhanced Protopanaxadiol Production in Saccharomyces cerevisiae
Source: Int J Mol Sci. 2021 Oct 31;22(21):11836. doi: 10.3390/ijms222111836 (PMC8584524; doi:10.3390/ijms222111836)
Supplement: Supplementary file 1 [file ijms-22-11836-s001.zip › ijms-1414316-supplementary.pdf]

CRISPRi-guided Metabolic Flux Engineering for Protopanaxadiol Production in *Saccharomyces cerevisiae*

Supplemental Material

Soo-Hwan Lim [1,a], Jong-In Baek [2,a], Byeong-Min Jeon [2], Jung-Woo Seo [1], Min-Sung Kim [2], Ji-Young Byun [2], Soo-Hoon Park[1], Su-Jin Kim[1], Ju Young Lee [4], Jun Hyoung Lee [1] and Sun-Chang Kim [1,2,3,*]

[1] Intelligent Synthetic Biology Center, 291 Daehak-Ro, Yuseong-Gu, Daejeon 305-701, Korea

[2] Department of Biological Sciences, Korea Advanced Institute of Science and Technology, 291 Daehak-Ro, Yuseong-Gu, Daejeon 305-701, Korea

[3] KAIST Institute for Biocentury, Korea Advanced Institute of Science and Technology, 291 Daehak-Ro, Yuseong-Gu, Daejeon 305-701, Korea

[4] Research Center for Bio-based Chemistry, Korea Research Institute of Chemical Technology (KRICT), 406-30, Jongga-ro, Jung-gu, Ulsan, 44429, Republic of Korea

[a] These authors contributed equally to this work.

*Correspondence: sunkim@kaist.ac.kr; Tel: +82-42-350-2619

Supplemental Table S1. Sequence, position of midpoint relative to start codon, and direction of ERG7 promoter-targeting sgRNAs used in this study.

Name	Sequence (5′ -> 3′)	Position of midpoint relative to start codon (bp)	sgRNA direction
sgRNA1	TCTTTGTGGGCGACGATTAT	−3	Antisense
sgRNA2	CGATTATTGGTAAGGTTATT	−36	Antisense
sgRNA3	CTGGAGAGGCTTGTGCTCAA	−74	Antisense
sgRNA4	CTTTTCGTTTATAATCGTTT	−186	Sense
sgRNA5	ACATATGGCTTGTTATGGTT	−425	Sense

Supplemental Table S2. Plasmids used in this study.

Plasmid	Source
pRS424TEF1	Addgene
pRS426GPD	Addgene
pRS426PGK1	Addgene
pTDH3-dCas9-Mxi1	Addgene
Cas9-NAT	Addgene
pRS42H	EUROSCARF
pRS42K	EUROSCARF
pRS424TEF1-ERG1m	This study
pRS426GPD-PgDS	This study
pRS426GPD-PgPPDS	This study
pRS426GPD-tHMG1	This study
pRS426PGK1-PgCPR	This study
pRS42H-sgRNA(GLK1)	This study
pRS42K-sgRNA(TEF2)	This study
pRS42K-sgRNA(RPS17B)	This study
pTDH3-dCas9-Mxi1-sgRNA1	This study
pTDH3-dCas9-Mxi1-sgRNA2	This study
pTDH3-dCas9-Mxi1-sgRNA3	This study
pTDH3-dCas9-Mxi1-sgRNA4	This study
pTDH3-dCas9-Mxi1-sgRNA5	This study

Supplemental Table S3. Primers used in this study.

Primer	Sequence (5′ to 3′)	Purpose
tHMGR1-BamHI-F	GACGGATCCATGGACCAATTGGTGAAAACTGAAGTCACCAA	Construction of pRS426GPD-tHMGR1
tHMGR1-XhoI-R	GACCTCGAGTTAGGATTTAATGCAGGTGACGGACCCAT	Construction of pRS426GPD-tHMGR1
SNR12p-EcoRI-F	GACGAATTCTCTTTGAAAAGATAATGTATGATTATGCTTTC	Construction of pTDH3-dCas9-Mxi1-sgRNAs
SUP4t-SpeI-R	GACACTAGTAGACATAAAAAACAAAAAAAGCACCACCG	Construction of pTDH3-dCas9-Mxi1-sgRNAs
sgRNA1-Insert-F	TCTTTGTGGGCGACGATTATGTTTTAGAGCTAGAAATAGCAAGTTAAAAT	Construction of pTDH3-dCas9-Mxi1-sgRNA1
sgRNA1-Insert-R	ATAATCGTCGCCCACAAAGAGATCATTTATCTTTCACTGCGGAG	Construction of pTDH3-dCas9-Mxi1-sgRNA1
sgRNA2-Insert-F	CGATTATTGGTAAGGTTATTGTTTTAGAGCTAGAAATAGCAAGTTAAAAT	Construction of pTDH3-dCas9-Mxi1-sgRNA2
sgRNA2-Insert-R	GAGTCAGGCTCTTGAAGCAGGATCATTTATCTTTCACTGCGGAG	Construction of pTDH3-dCas9-Mxi1-sgRNA2
sgRNA3-Insert-F	CTGGAGAGGCTTGTGCTCAAGTTTTAGAGCTAGAAATAGCAAGTTAAAAT	Construction of pTDH3-dCas9-Mxi1-sgRNA3
sgRNA3-Insert-R	TTGAGCACAAGCCTCTCCAGGATCATTTATCTTTCACTGCGGAG	Construction of pTDH3-dCas9-Mxi1-sgRNA3
sgRNA4-Insert-F	CTTTTCGTTTATAATCGTTTGTTTTAGAGCTAGAAATAGCAAGTTAAAAT	Construction of pTDH3-dCas9-Mxi1-sgRNA4
sgRNA4-Insert-R	AAACGATTATAAACGAAAAGGATCATTTATCTTTCACTGCGGAG	Construction of pTDH3-dCas9-Mxi1-sgRNA4
sgRNA5-Insert-F	ACATATGGCTTGTTATGGTTGTTTTAGAGCTAGAAATAGCAAGTTAAAAT	Construction of pTDH3-dCas9-Mxi1-sgRNA5
sgRNA5-Insert-R	AACCATAACAAGCCATATGTGATCATTTATCTTTCACTGCGGAG	Construction of pTDH3-dCas9-Mxi1-sgRNA5
TEF2-Integ-F	CCTCTTATTTTTATGTATTAGTTAATTAAGTATTTTTATCTATCTGCTTATTACGCCAAGCGCGCAATTAACCCTCACTAAAG	Integration of PgDS
TEF2-Integ-R	GTTTCTTTAGGTTTGATGAGGCCGTCTTTTGTTGATAGCAGCTTTTTCCAGGTACCGGCCGCAAATTAAAGCCTTC	Integration of PgDS
GLK1-Integ-F	CTGAGTTGGGCACTGATGGGGGGGTTGGGGGTTCTGTTGATAGCGGATATTTACGCCAAGCGCGCAATTAACCCTCACTAAAG	Integration of PgPPDS
GLK1-Integ-R	AAAACGGGAAATAACAATAACGACAAAAATGGAAAAAAAAAAAATTTTAGGCGTAATACGACTCACTATAGGGCGAATTG	Integration of PgPPDS
RPS17B-Integ-F	TATTATGGGCATTCCGAAGAATTTCATCTGGAAGACGCGCATTGATTGATTTACGCCAAGCGCGCAATTAACCCTCACTAAAG	Integration of PgCPR

RPS17B-Integ-R	TTCTCTAGGTTATACAACGCAAAATAAATAATTAATATGTAAAA TATCTGGCGTAATACGACTCACTATAGGGCGAATTG	Integration of PgCPR
TCB2-Integ-F	CGAGTGCTACTATAGTACTTTTCTTTATTACTACGTATGTAAATT GTAACTTACGCCAAGCGCGCAATTAACCCTCACTAAAG	Integration of tHMGR1
TCB2-Integ-R	TGGTGGTGTTTTAACTGTTGATGAGCTGTTTTGGTGGTTTAGC CTCCGTTAACTATGCGGCATCAGAGCAGATTGTACTG	Integration of tHMGR1
TRP1-Integ-F	AGGAAGCATTTAATAGAACAGCATCGTAATATATGTGTACTTT GCAGTTATGACGCCAGATGGCAGTAGTGGAAGATATTC	Integration of ERG1m
TRP1-Integ-R	TGTACAATCAATCAAAAAGCCAAATGATTTAGCATTATCTTTA CATCTTGTGATTACGCCAAGCGCGCAATTAACCCTCACTA	Integration of ERG1m
ISR1-Integ-F	CTAAAAGCTAAAGCAACAAGTTAATAAGGTTAGAAGATCGTA AATGTGTTTTACGCCAAGCGCGCAATTAACCCTCACTAAAG	Integration of dCas9-sgRNAs
ISR1-Integ-R	CTTTTTGTTTATTCAATGCTAGTCAAAATAAACAATAAGATAG ACCCATAAACTATGCGGCATCAGAGCAGATTGTACTG	Integration of dCas9-sgRNAs
ACT1-qRT-F	ACGTTCCAGCCTTCTACGTTTCCA	qRT-PCR
ACT1-qRT-R	ACGTGAGTAACACCATCACCGGAA	qRT-PCR
ERG7-qRT-F	ACCGTTTGACAAGATTAACTTCTCC	qRT-PCR
ERG7-qRT-R	TGGATAGAGAGTAAATGAACCG	qRT-PCR
dCas9-qRT-F	ACAAGAAGTATTCTATCGGACTG	qRT-PCR
dCas9-qRT-R	GATCAGATTTTTCTTGATGGAAT	qRT-PCR

Supplemental Table S4. Growth kinetic parameters of dCas9-sgRNA cassettes in metabolically engineered *Saccharomyces cerevisiae* strains.

Strains	$Y_{x/s}$ [$g \cdot g^{-1}$]	$Y_{p/x}$ [$mg \cdot g^{-1}$]	μ_{max} [h^{-1}]
PPD-A3	17.22 ± 0.86	0.08 ± 0.00	0.23 ± 0.01
PPD-A3-sgRNA1	16.51 ± 0.79	0.47 ± 0.03 *	0.21 ± 0.01 *
PPD-A3-sgRNA2	16.35 ± 0.79	0.97 ± 0.01 *	0.20 ± 0.01 *
PPD-A3-sgRNA3	14.15 ± 0.71 *	0.87 ± 0.00 *	0.16 ± 0.01 *
PPD-A3-sgRNA4	16.27 ± 0.79	1.17 ± 0.01 *	0.19 ± 0.01 *
PPD-A3-sgRNA5	16.43 ± 0.79	0.46 ± 0.01 *	0.22 ± 0.01

$Y_{x/s}$ = cell biomass yield based on substrate utilization, $Y_{p/x}$ = Protopanaxadiol yield based on substrate (D-glucose) utilization, μ_{max} = specific growth rate. Data are presented as means with standard deviation of biological triplicates. Statistical analysis was performed using Student's t-test (* $P < 0.05$).

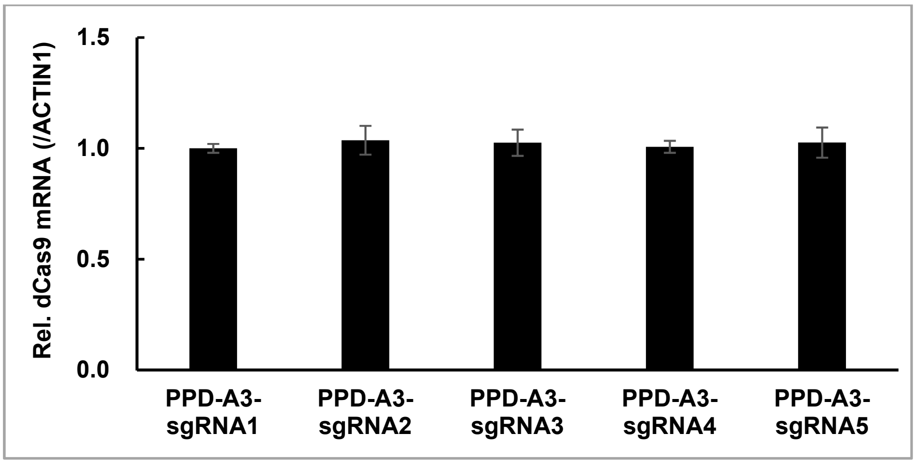

Supplemental Figure S1. Relative dCas9 mRNA levels in engineered yeast strains cultured for 48 h in YPD medium. Data are presented as mean and standard deviation of biological triplicates.
